# Simulation of a medical linear accelerator for teaching purposes

**DOI:** 10.1120/jacmp.v16i3.5139

**Published:** 2015-05-08

**Authors:** Rhys Anderson, Mike Lamey, Miller MacPherson, Marco Carlone

**Affiliations:** ^1^ Department of Medical Physics Trillium Health Partners Mississauga ON Canada; ^2^ Radiation Medicine Program Princess Margaret Cancer Centre Toronto ON Canada; ^3^ Department of Radiation Oncology University of Toronto Toronto ON Canada

**Keywords:** linear accelerator, simulation, teaching

## Abstract

Simulation software for medical linear accelerators that can be used in a teaching environment was developed. The components of linear accelerators were modeled to first order accuracy using analytical expressions taken from the literature. The expressions used constants that were empirically set such that realistic response could be expected. These expressions were programmed in a MATLAB environment with a graphical user interface in order to produce an environment similar to that of linear accelerator service mode. The program was evaluated in a systematic fashion, where parameters affecting the clinical properties of medical linear accelerator beams were adjusted independently, and the effects on beam energy and dose rate recorded. These results confirmed that beam tuning adjustments could be simulated in a simple environment. Further, adjustment of service parameters over a large range was possible, and this allows the demonstration of linear accelerator physics in an environment accessible to both medical physicists and linear accelerator service engineers. In conclusion, a software tool, named SIMAC, was developed to improve the teaching of linear accelerator physics in a simulated environment. SIMAC performed in a similar manner to medical linear accelerators. The authors hope that this tool will be valuable as a teaching tool for medical physicists and linear accelerator service engineers.

PACS number: 87.55Gh, 87.56bd

## I. INTRODUCTION & THEORY

Medical linear accelerators are commonly used in radiotherapy in order to create high‐energy MV radiation beams with well‐established therapeutic, practical, and radiation safety benefits. These devices are technologically complex. A good part of radiotherapy medical physics training programs is typically devoted to understanding the technical functionality of these devices, as well as the effects of machine adjustments on the therapeutic properties of the resulting X‐ray beams. Most radiotherapy departments employ specialized service personnel or dedicated service contracts with the manufacturer to effect regular maintenance and repairs on these devices.

Detailed descriptions of the radiation beam transport in the linear accelerator treatment head and patient have been developed, which has resulted in very high level of knowledge within the radiotherapy community of the dosimetric aspects of medical linear accelerator radiation beams. Less well developed is the basic understanding of how the adjustment of the linear accelerator's electrical components affects the resulting radiation beam. Other than the well‐known text by Karzmark et al.,^(1)^ there is very little literature devoted to the practical understanding of linear accelerator functionality targeted at the radiotherapy clinic level. While medical physicists typically receive a basic training on the high level principles of linear accelerator functionality, this training often lacks the hands‐on component that is required to develop a deeper understanding of basic electrical adjustments. This is often due to limited access to radiotherapy linacs that are in clinical operation, and more importantly, due to the fact that these machines typically can only be adjusted within a narrow set of parameters to maintain clinical operation.

Training for service personnel is often a major component early in their employment, since most service personnel learn the theory and practice of linac servicing while employed for this purpose. This initial “on‐the‐job” training can be lengthy and costly, and often requires special training from the manufacturers of medical linear accelerators.

Lacking in the radiotherapy community are tools to allow simple, low‐cost, hands‐on training of service personnel and medical physicists that can give a realistic understanding of the effect of basic electrical adjustments to medical linear accelerators. Other industries employ simulation software to improve access to training on complicated equipment and to reduce cost.^(2)^ Virtual tools do exist for the operation of linear accelerators;^3^, ^4^, ^5^, ^6^ however, these are aimed at operating linear accelerators. They do not simulate the mode of electron acceleration or photon production. Due to the complex physics of medical linear accelerators, simulation methods often employ computer‐intensive methods such as Monte Carlo (MC) and Finite Element Method (FEM) calculation techniques.^(7)^ While accurate, these tools cannot be used for simulation programs on typical desktop computers since they do not calculate beam effects in real time, rendering them ineffective for teaching purposes. Rather, they have been developed in order to simplify the design and manufacture of linear accelerator components, where speed of calculation is not often the primary objective.

The purpose of this work is to realistically simulate the effects of electrical adjustments pertaining to the dose rate of medical linear accelerators in real time using an ordinary personal computer and graphical user interface. We suggest that this new approach to linear accelerator simulation can have significant educational benefits to medical physicists and linear accelerator service personnel, but may also be useful to other professionals in radiotherapy, such as radiation oncologists and therapists. Further, the approach described can be implemented on simple and readily accessible computing equipment, and thus should be of low cost, improving accessibility to the entire radiotherapy community.

### A. Overview

To first and higher orders, all components of medical linear accelerator can be very well described by analytical solutions, which often provide very detailed and realistic descriptions of the basic electrical components of linear accelerators. When appropriate calibrations are applied, these analytical solutions can accurately simulate the performance of all linear accelerator subcomponents. Grouped together, an overall medical linear accelerator model can be constructed using simple analytical models that can be readily computed with low‐cost computers.

**Figure 1:**
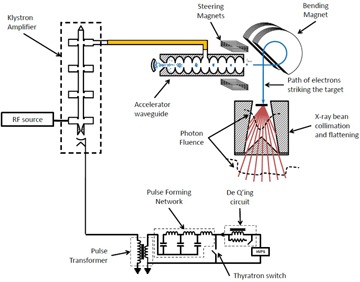
Diagram of linear accelerator system simulated in this work. A klystron amplifier is pulsed with high voltage created by discharge in a capacitor bank (pulse‐forming network), causing amplification of low‐power microwaves. This RF power is used to accelerate electrons injected into an accelerator waveguide, which can be steered by magnets. A bend magnet is used to redirect the high‐energy electrons onto a target, producing bremsstrahlung photons. These photons are processed in a collimation and flattening system to produce a clinical beam.

Currently available technology uses two similar, but technically different, approaches to medical linear accelerator design. One method employs a klystron amplifier to generate high power microwaves that are then used to accelerate electrons to megavoltage energies. Though not related to the choice of microwave amplification, this design typically uses a 270° style bending magnet to redirect electrons exiting the accelerator towards the linear accelerator isocenter. A second approach uses a magnetron oscillator to generate high‐power microwaves, which are then fed into a travelling wave accelerating waveguide. The travelling wave structure allows a microwave exit port to capture unused microwave power. This energy is then reinput into the accelerating waveguide to improve the energy gain of the accelerated electrons. For this second design, the method to redirect the electron motion towards the linear accelerator isocenter is to decompose the bending magnet into three components and arrange them in a manner that more closely resembles a 90° turn. As well, many other types of linear accelerator designs have been used in the past, such as the Varian 600 series and TomoTherapy linacs, which are magnetron‐powered and have no bend magnet. As well, there was the Siemens MD class of linear accelerator which was powered by a magnetron, but employed a 270° bend magnet. More recently, the TrueBeam class linear accelerator by Varian has a mode of operation without a flattening filter. All of these approaches provide high‐quality radiotherapy beams. In this work only the first method was simulated; however, there is no reason why the same approach could not be used for the other types of medical linear accelerator that are currently available commercially. Figure 1 shows a high level schematic diagram of the accelerator system modeled in this work. Medical linear accelerators can produce radiation beams comprising of either photons or electrons by employing a bremsstrahlung target or retracting it. In this simulation, we simulated linac photon mode only, since photons are more commonly used than electrons. Simulating photon mode requires the simulation of the target and flattening filter, where simulating electron mode requires simulation of a scattering foil and electron cone. In future versions of the software, we hope to also simulate electron mode.

The method used in this work is to group known analytical models of linear accelerator components together such that the overall model will describe to good precision the performance of a medical linear accelerator. These models are simple enough that they can be programmed in a MATLAB environment (MathWorks, Natick, MA), allowing a user interface similar to service screens of medical linear accelerators. Since the software was created to educate its users, interactive features were included in various capacities, such as the characteristic curves for a klystron amplifier and linac load line and oscilloscope traces of measureable electronic signals. Finally, simple dose distributions that would be measured in a conventional water phantom were also simulated.

To compartmentalize the software, it was divided into three natural partitions of a medical linear accelerator. The klystron amplifier section describes the nonlinear dependencies of the gain of this system in amplifying a radiofrequency electromagnetic pulse to high power. The accelerator section models the linear behavior of the output energy as a function of beam loading in the system, and the necessity of matching this energy with an appropriate magnetic field strength within the bending magnet. Lastly, the beam delivery system computes the variability of the dose rate and dose profile in a water phantom as the user varies the energy, current, or steering of the electron beam.

### B. Klystron amplifier

**Figure 2:**
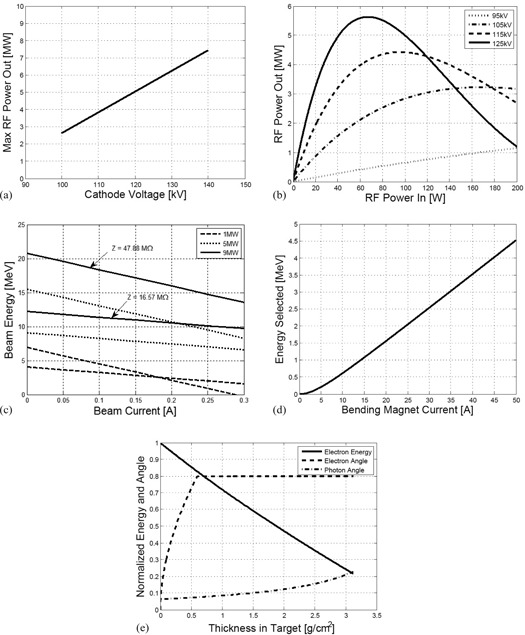
Maximum power output (a) from the klystron vs. peak voltage provided to the cathode. Power output (b) from the klystron vs. radiofrequency power driving it, for varying levels of cathode voltage. Beam energy (c) vs. beam current for two accelerator shunt impedances and three different klystron power levels input into the accelerator waveguide. For each of the three power levels, the largest slope load line has accelerator shunt impedance 47.88 MΩ, and the shallowest slope curve has shunt impedance of 16.57 MΩ. This is illustrated for the 9 MW curves. Average energy of electrons (d) accepted by the bending magnet versus current to the coils. Traces (e) of the mean electron energy (solid line), the root mean square electron scattering angle (dashed line), and the root mean square bremsstrahlung production angles (dashed‐dotted line), plotted as a function of thickness traversed through the target. The root mean square values were used to characterise the width of both Gaussian distributions.

The power output from the klystron was modeled mathematically as a function of two inputs from the user: the power of the radiofrequency signal to be amplified and the electric potential provided to the beam of electrons navigating the klystron. A simple theory of electron bunching in a two‐cavity klystron was employed to model the variation in power output as the radiofrequency power used to drive the system ($P_{\mathrm{RF}}$) changed. A suitable mathematical model for the power output from the system ($P_{\mathrm{Kly}}$) was found to be:^(8)^

(1) $$P_{Kly}=10P_{Max}{\left(\frac{J_{1}(X)}{X}\right)}^{2}\frac{P_{RF}}{P_{C}};\;X=1.84{\left(\frac{P_{RF}}{P_{C}}\right)}^{1/2}$$

where $P_{\mathrm{Max}}$ is the beam potential‐dependent maximum output power, and $P_{C}$ is the critical power level at which saturation occurred, and is a function of the beam potential. $J_{1}(X)$ is a Bessel function of first order. The maximum output power of a typical klystron, $P_{\mathrm{Max}}$, was found to obey a linear relationship over a wide range of typical beam potentials. For instance, the CPI Microwave Power Products CPI LLC, Palo Alto, CA) VKS‐8262 line of klystrons operates in this linear fashion for beam potentials from 100 kV to 145 kV according to a datasheet for this product.^(9)^ This linear relationship is plotted in Fig. 2(a). The overall amplification as described by Eq. (1) is plotted in Fig. 2(b).

### C. Accelerating waveguide

A defining feature of linear accelerator performance is that the energy gain of an electron beam can be modeled as an electric circuit where the RF source provides a voltage gain. The accelerator structure and electron beam are parallel impedances. The voltage gain of the accelerator structure is largely determined by its shunt impedance,^1^, ^10^, ^11^ which describes how RF power is converted to an accelerating potential. The accelerator beam can be modeled as a variable impedance which depletes energy from the microwave field to convert it to electron beam energy. The energy of the electrons emerging from the accelerator waveguide were modeled as decreasing linearly with the beam current, and had a no‐load energy that increased as the square root of the radiofrequency power input into the system from the klystron. The well‐known beam loading relationship for linear accelerators described as:^1^, ^10^, ^11^

(2) $$〈V_{Acc}〉=\sqrt{P_{Kly}Z}-\frac{i_{Acc}Z}{2}$$

where $V_{\mathrm{Acc}}$ is the beam potential, *Z* is the linear accelerator shunt impedance, $P_{\mathrm{Kly}}$ is defined as above, and $i_{\mathrm{Acc}}$ is the beam current. Example load lines are plotted in Fig. 2(c).

### D. Electron gun and bending magnet

The beam current in the accelerating waveguide, $i_{\mathrm{Acc}}$, is derived from inputs to a triode gun operating in a space‐charge limited modality. The Child‐Langmuir Law gives the relationship between the beam current produced by a Pierce‐type electron gun and the magnitude of the voltage at its cathode. It states that the maximum current produced grows as the three‐halves power of the voltage.^1^, ^12^ The beam current in the system was described as a function of the cathode voltage as:

(3) $$i_{Acc}=k_{1}\left(pV_{Cath}^{3/2}\right)\left(1-\frac{V_{Grid}}{V_{Cut}}\right)$$

where *p* is the perveance of the system, $V_{\mathrm{Cath}}$ is the voltage delivered to the cathode, $V_{\mathrm{Grid}}$ is the negative bias of the grid, and $V_{\mathrm{Cut}}$ is the cut‐off voltage, below which current ceases to flow, and $k_{1}$ is a proportionality constant^*^. Despite Eq. (3), grid gating effects are not described in the results below.

The distribution of beam energies emerging out of the accelerator and into the bending magnet was assumed to be Gaussian. The bending magnet only admitted electrons of energies within a certain range of its set‐point, and filtered out all others. Assuming the bending magnet to be a perfect solenoid, the average energy admitted through it was therefore given by the relativistic expression:

(4) $$E=〈E_{Mag}〉=\sqrt{{\left(k_{2}I_{Coil}\right)}^{2}+{\left(m_{2}c^{2}\right)}^{2}}-m_{e}c^{2}$$

where $k_{2}$ is a parameter that depended on the properties of the coil, and $I_{\mathrm{Coil}}$ is the current provided to the magnet.^(13)^ Figure 2(d) shows the nearly linear relationship between bending magnet current pass through energy of the bending magnet modeled in this work.

### E. X‐ray beam transport

The dose distribution calculated at a depth in water is a function of the average energy and average current of the beam as it strikes the target, as well as the angle and position. To first order, the dose rate at each point scales with average current delivered to the target, but also has a more complicated dependence on its incident position upon the target.^(14)^ To keep the simulation simple and within the scope of this work, only target current was used to model dose rate.

Steering magnets were modeled to allow changes to the position and angle at which the electron beam was directed onto the target. Both in‐plane and cross‐plane steering magnets were modeled; however, Fig. 1 shows only in‐plane steering magnets (to simplify the figure). The magnets themselves were modeled as simply deflecting the beam by an angle proportional to the steering current applied. The bending magnet was assumed to reproduce the phase space near the angle steering magnets at the target.^(1)^ In this manner, an electron leaving the accelerator at position ${\vec{r}}_{Acc}$ and angle ${\vec{\theta }}_{Acc}$ relative to the axis was predicted to have a position and angle at the target given by:

(5) $${\vec{r}}_{Tar}={\vec{r}}_{Acc}+d({\vec{\theta }}_{Acc}+\Delta {\vec{\theta }}_{Pos})$$

(6) $${\vec{\theta }}_{Tar}={\vec{\theta }}_{Acc}+\Delta {\vec{\theta }}_{Pos}+\Delta {\vec{\theta }}_{Ang}$$

where $\Delta {\vec{\theta }}_{Pos}$ and $\Delta {\vec{\theta }}_{Ang}$ are the deflections induced by the position and angle magnets respectively, and *d* is the length of the drift space between these two sets of magnetic coils. The phase space of the beam at the target was assumed to be a tight Gaussian about an average position and angle.

To determine the energy differential time‐averaged energy flux of X‐rays emerging from the target, henceforth referred to simply as the energy fluence spectrum, contributions from each layer of the target were summed. Findlay^(15)^ used the compiled tables of Scaled Bremsstrahlung Energy Spectra and Total Integrated Radiative Energy Loss Cross Sections^(16)^ to determine analytical relationships. He found that the data were well described by the expression:

(7) $$K\frac{d\sigma }{dK}=\frac{N_{A}}{A}Z^{2}a\left(1-b\frac{K}{E(x)}\right)$$

where *K* is the energy of the emitted photon^†^, E(x) is the mean kinetic energy of electrons at a thickness *x* in the target, $N_{A}$ is Avogadro's number, *Z* is the atomic number of the target material, *A* is its atomic mass, and α=11 mb and β=0.83 are linear regression parameters.

The mass stopping power as a function of energy S(E) was employed for the purpose of determining the mean kinetic energy of electrons within the target, E(x). This quantity was calculated via numerical evaluation of the expression:^(13)^

(8) $$E(x)=〈E_{Mag}〉-\int_{0}^{x}S(E(x^{\prime }))dx^{\prime }$$

Values of S(E) were determined from interpolation of data provided by NIST.^(17)^

The angular distribution of photons emitted at each layer in the target was considered separable from the bremsstrahlung photon energy cross section.^(15)^ The root mean square angle of bremsstrahlung photon emission was taken to be:^(18)^

(9) $$\sigma_{b}=\sqrt{〈\theta_{b}{}^{2}〉}=k_{3}\frac{m_{e}c^{2}}{E(x)+m_{e}c^{2}}$$

where $k_{3}$ is a factor modified to fit the expected distribution. The angular distribution of each of these bremsstrahlung photons was assumed to be Gaussian with respect to the angle of incidence of the incident electron.^(15)^ These two approximations were justified based on inspection of the small‐angle approximation to the Schiff cross section.^(19)^

Furthermore, the distribution in angle of electron trajectories at a thickness *x* in the target was given by Molière's theory of multiple electron scattering to be approximately Gaussian^20^, ^21^ with root mean square angle:

(10) $$\sigma_{e}=\sqrt{〈\theta_{b}{}^{2}〉}=\frac{13.6MeV}{E_{0}+m_{e}c^{2}}{\left(\frac{x}{X_{0}}\right)}^{1/2}\left(1+0.038\;\text{ln}\frac{x}{X_{0}}\right)$$

where $X_{0}$ is the radiation length of the target material^(22‐24^) and $E_{0}$ is the initial kinetic energy of the electron striking the target. The root mean square angle given in the above expression was found to saturate at 0.8 radians, at which point the electrons were completely dispersed in the target.^(25)^ Figure 2(e) shows a plot of the change in root mean square angle and mean electron energy through the target for incident 6 MeV electrons.

Combining the above distributions, the average energy fluence spectrum from the target was calculated to be:^(15)^

(11) $$\frac{d〈\Psi 〉}{dK}(K,\theta )=〈i_{Tar}〉\int_{0}^{t}e^{-\frac{\mu (K)}{\rho }(t-x)}\frac{N_{A}Z^{2}}{A}a\left(1-b\frac{K}{E}\right)\frac{1}{\pi (\sigma_{e}{}^{2}+\sigma_{b}{}^{2})}e^{-\frac{{\left(\theta -\theta_{0}\right)}^{2}}{\sigma_{e}{}^{2}+\sigma_{b}{}^{2}}}dx$$

where *t* is the target thickness, $\theta_{0}$ is the magnitude of the initial angle of the incident electrons with respect to the target normal, *K* is defined as in Eq. (7), and $\frac{\mu (K)}{\rho }$ is the energy‐dependent, mass‐attenuation coefficient of the photons in the target.^(26)^

### F. Beam shaping and measurement model

The flattening filter and collimators provided attenuation of the X‐ray distribution produced by the target, as illustrated in Fig. 1. The initial average irradiance as a function of angle was transformed to a function of space based on the initial position at which the electron beam was incident on the target, and the drift space between important elements of the beam delivery system. Collimators were assumed not to transmit any energy flux incident upon them. The flattening filter provided X‐ray attenuation dependent on the photon energy and its position‐dependent thickness. For an average irradiance $\frac{\mu (k)}{\rho }$ incident on the fattening filter, the average irradiance emitted was modeled by the expression:

(12) $$\frac{d〈\Psi (x,y,z=d_{f}^{+})〉}{dK}=e^{-\frac{\mu (K)}{\rho }t(x,y)}\frac{d〈\Psi (x,y,z=d_{f})〉}{dK}$$

where $d_{f}$ is the distance from source to filter, and t(x,y) is the function for the thickness of the filter at positions *x* and *y* in the plane of the target. The model was based on gold standard data available from Varian Medical Systems (Palo Alto, CA).^27^, ^28^

For depths greater than that of the maximum dose, the time‐averaged dose rate was approximated as being equal to the time‐averaged kerma.^(26)^ Thus, the expression for dose rate was:

(13) $${〈D^{\prime }(x,y,d_{SAD})〉}_{t}=\int_{0}^{v}e^{-\frac{\mu (K)}{\rho }\delta }\frac{d〈\Psi (x,y,d_{SAD})〉}{dK}\frac{\mu_{ab}(K)}{\rho }dK$$

where $\frac{\mu_{ab}(K)}{\rho }$ are the mass‐absorption coefficients and are obtained from the NIST database,^(29)^ and $d_{\mathrm{SAD}}$ is the distance to the isocenter.

A scaled convolution was performed between the calculated dose distribution and the spot size in order to account for first‐order corrections due to the spot size being finite, which included the prediction of a geometric penumbra. The final dose rate was therefore given by the expression:

(14) $${〈D(x,y,d_{SAD})〉}_{t}=d_{f}\iint \frac{{〈D^{\prime }(x-u,y-v,d_{SAD})〉}_{t}}{d_{SAD}-d_{f}}f\left(\frac{d_{f}u}{d_{SAD}-d_{f}},\frac{d_{f}v}{d_{SAD}-d_{f}}\right)dudv$$

Here *f* is a two‐dimensional Gaussian distribution function describing the initial positions of electrons striking the target, where the scaled *x* and *y* coordinates are given by $\frac{d_{f}u}{d_{SAD}-d_{f}}$ and $\frac{d_{f}v}{d_{SAD}-d_{f}}$.

## II. MATERIALS AND METHODS

**Figure 3:**
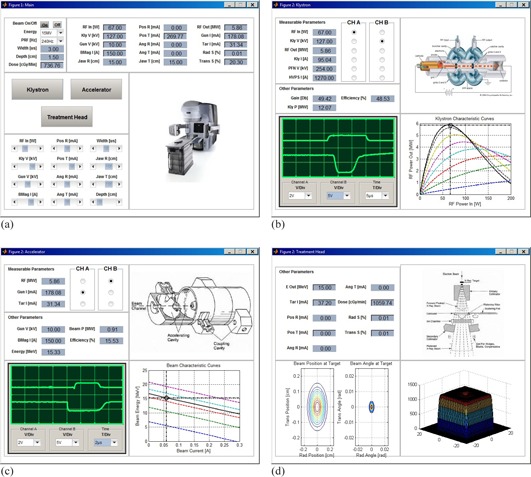
SIMAC screen shots: (a) the principal screen [Image courtesy of Varian, with permission]; (b) the Klystron screen, which includes plots of Eq. (1); (c) the linac screen, which includes load line plots, Eq. (2) [Waveguide image from Karzmark et al.^(1)^, with permission from McGraw‐Hill Education] (d) the treatment head view, showing the effects of beam steering on dose profiles in water [Treatment head image from Karzmark et al.^(1)^, with permission from McGraw‐Hill Education].

The expressions contained in (1), (2), (3), (4) and (5), (6), (7), (8), (9), (10), (11), (12), (13), (14) were coded using MATLAB v. 7.14. The program was called SIMAC, which is meant to abbreviate the phrase “Simulate Linac.” When launched, SIMAC produces a main screen where four linac electrical parameters can be input (see Fig. 3(a)). These inputs are the RF driver output power (RF In), the klystron voltage (KLY V), the accelerator gun voltage (Gun V), and the bending magnet current (BMAG I). Parameters that affect beam steering can also be adjusted. The program allows the user to enter preset parameters for 6 and 15 MV beams. Choosing either energy also selects a flattening filter optimized for that energy. The main window also displays the resulting linac performance in terms of dose rate and other measurable operating characteristics: klystron RF output power, gun current, and target current, as well as in‐plane and cross‐plane beam symmetry. Table 1 lists all the parameters used in SIMAC and gives a brief description.

**Table 1:** Summary of parameters used in SIMAC.

*SIMAC Parameter*	*Meaning*	*Units*
Energy	X‐ray beam energy	MV
PRF	Pulse repetition frequency	Hz
Width	Beam pulse width	μs
Depth	Depth in water phantom for beam analysis	cm
Dose Rate	The beam dose rate	cGy/min
RF In	RF power input to the klystron	W
RF Out	RF power exiting the klystron	MW
Kly V	Voltage of pulse to klystron cathode	kV
Kly I	Current of klystron pulse	A
Gun V	The voltage of the gun cathode pulse	kV
Gun I	The current of the gun cathode pulse	mA
Tar I	The beam current reaching the target	mA
BMag I	The bending magnet current	A
Jaw R	The jaw size in the in‐plane direction	cm
Jaw T	The jaw size in the cross‐plane direction	cm
Pos T	The current in the steering coil closest to the exit of the waveguide which moves the beam in the cross‐plane direction.	A
Pos R	The current in the steering coil closest to the exit of the waveguide which moves the beam in the in‐plane direction.	A
Ang T	The current in the steering coil furthest from the exit of the waveguide which moves the beam in the cross‐plane direction.	A
Ang R	The current in the steering coil furthest from the exit of the waveguide which moves the beam in the in‐plane direction.	A
Rad S	Radial (in‐plane) symmetry	%
Trans S	Transverse (cross‐plane) symmetry	%
PFN V	Voltage on the PFN (Pulse Forming Network)	kV
HVPS I	High voltage power supply current	A

Figure 3 shows SIMAC's graphical user interface, which was designed to simulate linear accelerator adjustments that would be done when repairing or servicing the unit. Sliding bars were used to change inputs to the program, and readout panels were used to show the effect of input changes on the operating point of the linear accelerator. As well, readouts are shown in the form of oscilloscope traces, which are typically used to monitor linear accelerator internal operation. From the main program window, three other windows can be opened. The first is the “klystron” window, which displays parameters required to set the klystron mode of operation, such as RF driver power and klystron pulse voltage. Graphs of the klystron gain as a function of klystron cathode pulse voltage are also shown on this window. The second window is the “accelerator” window, which displays accelerator beam parameters: RF input power, gun and target current, gun voltage, and bending magnet current. A graph of the accelerator load line is also shown, as well as the operating point on this curve and the resulting electron energy. The final window is the “treatment head” window which displays currents in steering coils, as well as graphics illustrating the electron beam position and angle of incidence on the target. A 3D beam fluence profile is also displayed.

## III. RESULTS

A systematic approach was used to evaluate the performance of the SIMAC program. An operating point with energy of 15 MV and dose rate of 420 cGy/min was simulated. The parameters that most affect beam energy, RF input power to the linac and the gun current, were independently varied and their effect on dose rate and beam energy was recorded.

**Figure 4:**
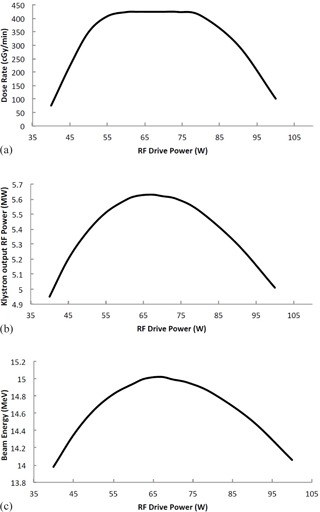
Dependence of (a) Klystron output power, (b) dose rate, and (c) beam energy on the RF driver power setting. Klystron pulse voltage was fixed at 125 kV, bending magnet current was fixed at 150 A, and the linac gun voltage was held at 10 V.

Figure 4(a) shows how Klystron output power is affected by the RF drive power input into it. Klystron's exhibit what is called “saturation,” where the klystron output power will increase in a linear fashion at low RF drive powers. At a certain point, the klystron output power maximizes and eventually decreases. The power drop‐off is due to dephasing of electron bunches within the klystron as the initial acceleration is too large and pushes or pulls electrons out of phase with the bunch, which results in a power drop‐off. Our model, Eq. (1), demonstrates this nicely for the model of klystron that we modeled (CPI model 8262) as seen in Fig. 4(a). Figure 4(b) shows the effect or RF drive stability on beam energy and linac dose rate. In these figures, the RF drive power to the klystron was varied from 40 to 100 W, as done in Fig. 4(a), while keeping other parameters fixed.

**Figure 5:**
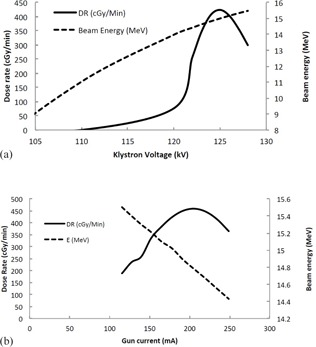
Dose‐rate dependence on klystron pulse voltage (a) and accelerator gun current (b). On the left, the RF driver power was fixed at 67 W, the linac gun voltage was set to 10 kV, and the bending magnet was left at 150 A. On the right, the RF driver was set to 67 W, the klystron pulse was 125 kV, and the bending magnet 125 A. The linac gun voltage was varied from 7.5 V to 12.5 V, which resulted in gun currents ranging from 115 to 249 mA and variability in dose rate. The electron beam energy is also affected by accelerator current due to beam loading.

The dose‐rate dependence on RF power was also verified by adjusting the klystron pulse voltage and linac gun current while keeping other linac parameters constant. Figure 5 shows the effect of klystron voltage on dose rate. Increasing klystron pulse voltage increases dose rate, since the higher RF power output from the klystron produces a larger accelerating voltage in the waveguide, which in turn produces a more energetic electron beam which increases the dose per pulse. However, dose rate eventually decreases as the RF power is further increased due to a mismatch between the linac beam energy and the acceptance energy of the bending magnet. The effect of changing accelerator beam current is also shown in Fig. 5. In this case, the increased beam current leads to a higher dose per pulse since the target current is also increasing. However, the energy of the electron beam decreases as the beam current is increased due to beam loading (Eq. (3)), and so the dose rate eventually maximizes and then decreases as beam current is further increased.

**Figure 6:**
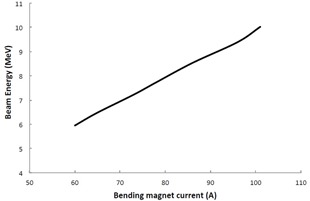
Characteristic curve of the bending magnet, showing electron beam acceptance of 1 MeV per 10 A increment in bending magnet.

By adjusting the RF power and accelerator beam current over a wide range, it was possible to simulate the response of the bending magnet to a large change in electron beam energy. Starting with a beam current of 6 MeV, RF power and beam current were adjusted in small increments to increase this energy to 10 MeV. Small increments are the preferred method to make this energy change both in practice and when using SIMAC. Because of the narrow admittance of the bending magnet, a change in beam energy will cause fewer electrons to reach the target, since the beam path is no longer matched to the bending magnet. Adjusting the bend magnet current to realign the beam path reestablishes the target current. This is important in practice, since the bending magnet is not usually designed to absorb a large beam current (power), and so the practice of using small increments with little drop in dose rate ensures the bend magnet will not be exposed to a direct electron beam. In SIMAC, small increments are also useful since they allow the user to keep a beam reference and not lose the beam in the multiparameter space that SIMAC allows. Figure 6 shows the change in bending magnet current required to maximize dose rate as the beam energy was increased. This figure shows a linear relationship that is consistent with Eq. (4), where a 10A/MeV calibration was applied.

**Figure 7:**
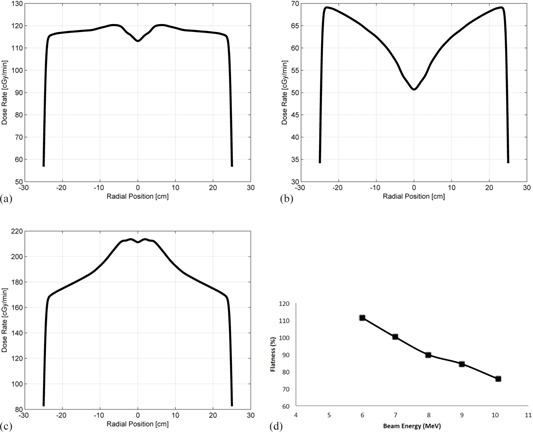
Beam profiles for different beam energies on the same flattening filter: (a) 6 MeV electron beam incident on 6 MV flattening filter; (b) 4.5 MeV electron beam incident on 6 MV flattening filter; (c) 7.5 MeV electron beam incident on 6 MV flattening filter; (d) flatness at depth 1.5 cm as a function of incident electron beam energy for a 6 MV flattening filter. Flatness was defined as the ratio of the dose rate at 3/4 jaw setting to central axis dose rate.

The systematic testing described in the previous paragraph also allowed simulation of the response of a single flattening filter to large changes in beam energy. (12), (13), (14) model the photon production in the plane perpendicular to the electron beam incident on the target, as well as photon attenuation in the beam collimator area and in a water‐equivalent phantom. We used the results of this to estimate the effect of changing energy on beam flatness. The results thus predicted by these equations are shown in Fig. 7, which shows beam profiles for electron beams of different energies incident on a fixed target/flattening filter combination, as well as a plot of flatness as a function of electron beam energy for a fixed flattening filter. This curve shows the flatness decreasing sharply, as beam energy is increased. This is expected, as more energetic beams have higher bremsstrahlung production along the direction of electron incidence. Thus beam profiles will become less flat and approach those of flattening filter‐free (FFF) beams as energy is increased to the extent simulated in Fig. 7. For a more comprehensive discussion of beam fatness dependence on energy, the reader may consult Gao and colleagues.^(30)^

**Figure 8:**
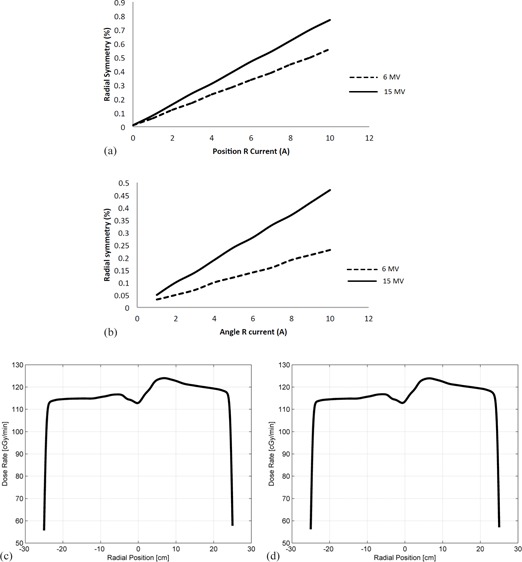
Beam symmetry as a response to steering coils current variation. Currents in coils meant to simulate Varian style accelerators with angle and position coils were varied, showing a linear response to beam symmetry: (a) position coil symmetry response; (b) angle coil symmetry response; (c) beam profile with coil currents Pos R=0, Ang R=100 mA; (d) beam profile with coil currents PosR=100 mA, AngR=0. The profiles in plots (c) and (d) appear almost identical; however, the reader will notice a very slight position change in the negative direction for plot (d) with PosR=100 mA. This shows that the position coil affects both the beam position on the target, but also its angle of incidence.

The response of accelerator beam steering was modeled using (5), (6) for steering magnets, and (7), (8), (9), (10), (11), (12), (13), (14) for bremsstrahlung production and photon transport. Figure 8 shows the simulated performance of the steering magnets for different beam energies and some resulting beam profiles.

## IV. DISCUSSION

The purpose of the SIMAC program is to simulate the functionality of a linear accelerator and to give the correct “feel” of adjusting linac service parameters. It is not intended to produce an exact response, but it is expected that the response be representative of the performance of a functioning medical linear accelerator so that it can be used in an educational environment for teaching purposes. To meet these requirements, the SIMAC program was optimized to respond in real time by compromising on the complexity of the model employed without sacrificing the type of response typical linear accelerators would produce. Exact mathematical models of linear accelerator physics are complicated, and often counterproductive, in teaching the practical aspects of medical linear accelerator maintenance, since the physical expressions do not relate directly to quantities that have meaning in maintaining the clinical aspects of linear accelerator beams. The most important examples of this are the linear accelerator shunt impedance (Eq. (2)) and diode electron gun perveance (Eq. (3)). Simply put, the shunt impedance reflects the ability of an accelerating waveguide to convert RF energy into an accelerating potential applicable to the linear acceleration of an electronic beam. It is a complicated function of accelerator cavity design and depends strongly on quantities such as RF frequency, phase shift per cavity, cavity coupling design (side couple coupling vs. on‐axis coupling), and the resulting concentration of magnetic and electric energy within an accelerator cavity.^10^, ^11^ For an exact description of linear accelerator shunt impedance, detailed calculations of electromagnetic field patterns based on waveguide theory are required.^1^, ^31^ While required for precise and detailed modeling of a medical linear accelerator, this type of detail is largely lost when translating that functionality to the more practical level service adjustments on a working linear accelerator.

The linear relation between the energy gain in an accelerating waveguide and beam current given by the shunt impedance relation, Eq. (2), has been extensively explored and supported by theory as well as experiment.^(11)^ To be useful a simulation program, such as SIMAC, correct calibrations of parameters such as shunt impedance, perveance, and others are required in order to use parameters that will reflect realistic linac functionality. In our experience, suitable parameters can be found in the literature, from manufacturers, or by simple experiments on functioning linear accelerators that kept the operating parameters with the clinical range.

Medical linear accelerators fail either catastrophically, where subcomponents stop functioning and need to be replaced, or by drifting away from a preset operating point. As well, medical linear accelerators may need large scale adjustments at the time of commissioning, but require only small scale adjustments for routine service and quality control. In either circumstance, fine tuning of control voltages is typically required to match the performance of a new component to the one that failed, or to bring a component whose output has drifted back to a value established when the operating model of the linear accelerator was established, either at the time of commissioning or subsequent quality control. This practice is often referred to as “Beam Tuning” and has the large‐scale effect of matching the operating point of the linear accelerator with that of the bending magnet. By “matching” we mean a match of the output electron energy of the linear accelerator with the pass‐through energy of the bending magnet at a beam current which produces a desired dose rate. Once this match has been set at initial linear accelerator setup and commissioning, any drift of control voltages to the linac beam control system typically produces a decrease in dose rate due to linac‐bending magnet mismatch. Figure 5 shows the macro effect of different strategies of beam tuning, where the goal is to adjust microwave power and beam current such that the energy of the electron beam is matched to the pass‐through energy of the bending magnet at the correct dose rate. These two methods of beam tuning are what are most often done in practice. They depend on a stable klystron operating point, as shown in Fig. 4. Variable RF driver amplification has a complicated effect on the beam's energy and dose rate, since the RF drive input power affects the RF power input into the linear accelerator, which in turn affects the beam energy and dose rate. In practice, to achieve a stable beam point, the klystron amplifier will be operated at saturation, as shown in Fig. 4(b). This figure shows that, for RF drive powers between about 55 to 80 W, a very stable dose rate can be achieved since changes in RF drive power in this range have relatively little effect on output klystron power, which in turn produces a stable beam.

In order to understand and learn the strategy and techniques required for these adjustments, supervised access to medical linear accelerators is required to ensure that any adjustments do not harm the linear accelerator. It is possible to direct the electron beam towards components that are not designed to absorb an electron beam of significant power by incorrect voltage adjustments. For instance, the bending magnet chamber, required to keep the electron flight path under vacuum, is typically not constructed to absorb electron beam energy, so care is required to avoid damaging this component during beam adjustments, which usually necessitates a supervised learning environment. Safe beam adjustments are typically done in small control voltage increments, and so there is often a barrier to understanding and teaching the gross effect of large parameter adjustments on the subsequent operation of the medical linear accelerator. The simulation software described here provides an opportunity to overcome this in a safe and economical manner. It also facilitates the collaboration required between medical physicist and service engineer, since it allows a common environment where the two can adjust linear accelerator beam parameters together and discuss their meaning in terms of clinical beam quality without the added constraints of time pressure and other clinical factors. As an example, Fig. 7 shows the simulated beam profiles for a very wide range of incident electron energies on a fixed target/flattening filter combination, illustrating the energy dependence of beam flatness. From an education point of view, this has benefit; however, because of the risk to the linac, it is difficult, if not impossible, to demonstrate this phenomena on an operating clinical linac. We suggest that SIMAC has value in teaching and demonstrating this type of accelerator physics.

The physical models used in this work represent a minimum required to simulate linear accelerator functionality. We believe it is possible to expand the physical models in a simple manner consistent with first‐order approximations that would expand the simulation package to include other serviceable concepts for medical linear accelerators. Equation (3) allows for a triode gun by including a grid voltage. We did not model a triode gun in this work; however, they are widely used in medical linear accelerators and have the advantage of allowing the control of the electron gun total charge per pulse, independently of the RF power applied to the linear accelerator waveguide. As well, temperature effects were not modeled in this work. The temperature of the linear accelerator is tightly controlled in practice since temperature changes produce changes in the size the RF cavities within the waveguide, which in turn changes its operating frequency and impedance at a fixed frequency. A rule of thumb is that 1° Celsius temperature change produces a resonant frequency shift of 50 kHz. Using this as a first‐order effect, it would be relatively simple to expand SIMAC to include the effects of temperature drift and to control these using a simulated automatic frequency control system (AFC). As well, ion chambers were not modeled in this work, so servo circuits for beam steering were not modeled. We hope to be able to address these and other model improvements in future versions of the software.

The expected value of SIMAC is as a teaching tool for both medical physicists and linear accelerator service personnel. Lacking in the clinical setting is a common language between accelerator service and physicists where consequences of serviceable adjustments are simply discussed in terms of clinical beam effects. Prudence and a heightened culture of caution imply that measurements of the clinical properties of photon and electron beams be done after linear accelerator servicing. In many cases this is justified; however, the authors are aware of situations where physicists without an appreciation for the meaning of some simple linear accelerator servicing have performed excessive quality assurance in order to ensure clinical operation. A common learning tool for physicists and linear accelerator service personnel would allow common language and common understanding of the risks of certain service procedures and thus better assessment of the cost benefit of linear accelerator quality control. Since the primary purpose of the software is for education, it is important to emphasize that the parameters used in the software are not representative of the actual parameters employed in working medical linear accelerators. We have taken care to apply appropriate constants and scaling factors in the models employed. The result is that realistic parameters are used by the program, which we feel is helpful in a teaching environment. However it must be strongly emphasized that SIMAC cannot be used to determine the operating point of clinical linacs.

The long‐term goal of the SIMAC project is to develop a freely available resource that can be used by anyone interested in learning about linear accelerator physics or by other people who would like to contribute to it and improve it. The long‐term vision is for the software to be distributed in an unrestricted manner for use in teaching and research, much like other medical physics codes such as EGSnrc (http://www.nrc‐cnrc.gc.ca/eng/solutions/advisory/egsnrc_index.html) and 3D Slicer (http://www.slicer.org/). However, the development environment used, MATLAB, does not readily lend itself to distribution and improvements. Stand‐alone versions are possible, but do now allow the user to improve the software unless he/she has access to MATLAB. Further improvements to the project include technical improvements, but also equally important, we wish to convert the software to a Web‐based environment which can be distributed more easily and facilitate collaborations among users.

## V. CONCLUSIONS

A simulation software, SIMAC, has been designed and built to allow real‐time simulation of medical linear accelerator service adjustments. The program can be run on any personal computer, and it has been shown to function and respond similarly as actual linear accelerators when its functionality was analyzed in a systematic fashion. The authors expect that this simulation software will be useful in teaching linear accelerator physics to medical physicists and service engineers, and it is hoped that this common learning environment would produce better collaboration between these two groups. The software employs simple analytical models of the physical phenomena of linear accelerators components, and can be expanded to more complex phenomena in future versions of the software.
